# 8-Acetyl-4-methyl-2-oxo-2*H*-chromen-7-yl acetate

**DOI:** 10.1107/S1600536811048835

**Published:** 2011-11-19

**Authors:** Shu-Ping Yang, Li-Jun Han, Xin-Ran He, Li-Juan Chen

**Affiliations:** a Department of Chemical Engineering, Huaihai Institute of Technology, Lianyungang 222005, People’s Republic of China; b Department of Mathematics and Science, Huaihai Institute of Technology, Lianyungang 222005, People’s Republic of China

## Abstract

In the title compound, C_14_H_12_O_5_, the benzopyran-2-one ring system is approximately planar [maximum deviation = 0.018 (1) Å]; the mean plane is oriented at dihedral angles of 52.26 (11) and 72.92 (7)°, respectively, to the acetyl and acet­oxy groups. In the crystal, π–π stacking is observed between parallel benzene rings of adjacent mol­ecules, the centroid–centroid distance being 3.6774 (17) Å. Inter­molecular weak C—H⋯O hydrogen bonding, and C=O⋯C=O [O⋯C = 3.058 (3) Å] and C=O⋯π [O⋯centroid = 3.328 (2) Å] inter­actions occur in the crystal structure.

## Related literature

For structures of related coumarin derivatives, see: Yang *et al.* (2006 ▶, 2007 ▶, 2010 ▶).
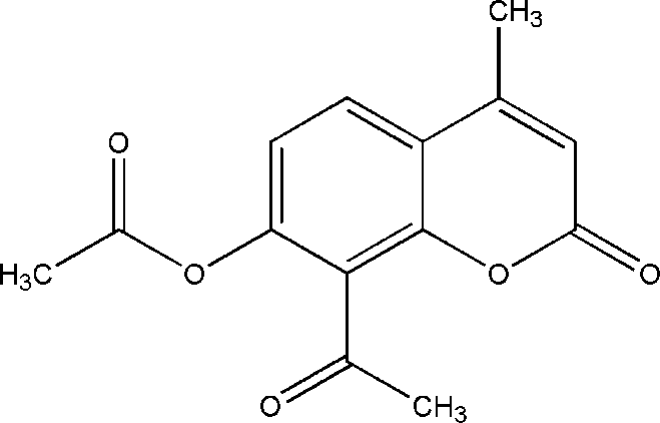


## Experimental

### 

#### Crystal data

C_14_H_12_O_5_
*M* *_r_* = 260.24
Triclinic, 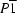

*a* = 8.198 (3) Å
*b* = 8.504 (3) Å
*c* = 9.644 (3) Å
α = 90.213 (4)°
β = 97.761 (4)°
γ = 111.686 (4)°
*V* = 618.0 (3) Å^3^
*Z* = 2
Mo *K*α radiation
μ = 0.11 mm^−1^
*T* = 298 K
0.30 × 0.10 × 0.10 mm

#### Data collection

Bruker APEXII CCD area-detector diffractometer
Absorption correction: multi-scan (*SADABS*; Bruker, 2001 ▶) *T*_min_ = 0.969, *T*_max_ = 0.989
4891 measured reflections
2292 independent reflections
1572 reflections with *I* > 2σ(*I*)
*R*_int_ = 0.030

#### Refinement

*R*[*F* ^2^ > 2σ(*F* ^2^)] = 0.048
*wR*(*F* ^2^) = 0.150
*S* = 1.07
2292 reflections
175 parameters
H-atom parameters constrained
Δρ_max_ = 0.24 e Å^−3^
Δρ_min_ = −0.19 e Å^−3^

### 

Data collection: *APEX2* (Bruker, 2007 ▶); cell refinement: *SAINT* (Bruker, 2007 ▶); data reduction: *SAINT*; program(s) used to solve structure: *SHELXS97* (Sheldrick, 2008 ▶); program(s) used to refine structure: *SHELXL97* (Sheldrick, 2008 ▶); molecular graphics: *DIAMOND* (Brandenburg & Berndt, 1999 ▶); software used to prepare material for publication: *SHELXL97*.

## Supplementary Material

Crystal structure: contains datablock(s) I, global. DOI: 10.1107/S1600536811048835/xu5385sup1.cif

Structure factors: contains datablock(s) I. DOI: 10.1107/S1600536811048835/xu5385Isup2.hkl

Supplementary material file. DOI: 10.1107/S1600536811048835/xu5385Isup3.cml

Additional supplementary materials:  crystallographic information; 3D view; checkCIF report

## Figures and Tables

**Table 1:** Hydrogen-bond geometry (Å, °)

*D*—H⋯*A*	*D*—H	H⋯*A*	*D*⋯*A*	*D*—H⋯*A*
C6—H6⋯O2^i^	0.93	2.52	3.324 (3)	145

Symmetry code: (i) 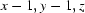.

**Fig. 1.**
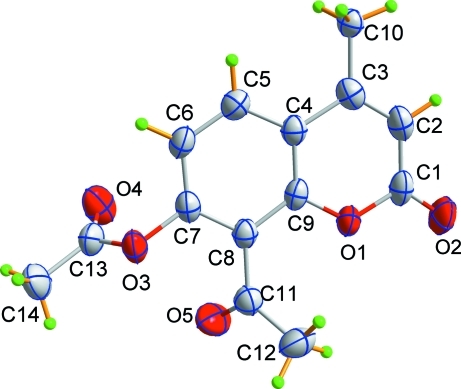
The molecular structure of the title compound, showing the atom-numbering scheme. Displacement ellipsoids are drawn at the 50% probability level.

## supplementary crystallographic information

### Comment

Previous we have reported the crystal structures of some coumarin derivatives (Yang *et al.*, 2006, 2007, 2010). As part of our study of the crystal structures of coumarin derivatives, we decribed here the crystal structure of 8-acetyl-7-acetoxy-4-methyl-2*H*-1-benzopyran-2-one, (I).

In the molecule (I) (Fig. 1), the benzopyran-2-one ring system is approximately palnar [maximum deviation 0.018 (1) Å]; the mean plane is oriented with respect to the acetyl and acetoxy groups at 52.26 (11) and 72.92 (7)°, respectively.

Molecules are linked together by one weak C—H···O hydrogen bond (Table. 1), two C═O···C═O interactions: C1═O2···C13^ii^═O4^ii^ [O2···C13^ii^ = 3.101 (3) Å, C1═O2···C13^ii^ = 143.37 (15)°; symmetry code:(ii). *x* + 1, *y* + 1, *z*] and C1═O2···C1^iii^═ O2^iii^ [O2···C1^iii^ = 3.058 (3) Å, C1═O2···C1^iii^ = 88.63 (13)°; symmetry code: (iii). 1 - *x*, 2 - *y*, 2 - *z*.], one C═ O···π interaction C1═O2···*Cg*1^iii^ [O2···*Cg*1^iii^ = 3.328 (2) Å, C1═O2···*Cg*1^iii^ = 113.74 (14)°, *Cg*1 is the centroid of the pyran ring] and one π-π stacking interaction *Cg*2···*Cg*2^iv^ [*Cg*2···*Cg*2^iv^ = 3.6774 (17) Å, *Cg*2 is the centroid of the benzene ring; symmetry code: (iv) -*x*, 1 - *y*, 2 - *z*] and generated a two-dimensional crystal structure by translation and inversion symmetry.

### Experimental

To a solution containing 8-acetyl-7-hydroxy- 4-methylcoumarin (2.18 g, 10 mmol) and anhydrous pyridine (10 ml), a solution of acetic anhydride (1.53 g, 15 mmol) was slowly added at 278–283 K, with stirring, then the reaction mixture was stirred at 303 K continuously for 24 h and then poured into ice–water (200 ml). The solid obtained was filtered off, washed with water and dried at room temperature. Colourless crystal suitable for X-ray structure analysis were obtained by recrystallizing the crude product from an ethanol solution [m.p. 484–485K].

### Refinement

All H-atoms were placed in calculated positions (C—H = 0.93 and 0.96 Å) and were included in the refinement in the riding model, with *U*_iso_(H) = 1.2*U*_eq_(aromatic C) and *U*_iso_(H) = 1.5*U*_eq_(methyl C).

### Crystal data

**Table:**

C_14_H_12_O_5_	*Z* = 2
*M**_r_* = 260.24	*F*(000) = 272
Triclinic, *P*1	*D*_x_ = 1.399 Mg m^−^^3^
Hall symbol: -P 1	Mo *K*α radiation, λ = 0.71073 Å
*a* = 8.198 (3) Å	Cell parameters from 1283 reflections
*b* = 8.504 (3) Å	θ = 2.6–25.2°
*c* = 9.644 (3) Å	µ = 0.11 mm^−^^1^
α = 90.213 (4)°	*T* = 298 K
β = 97.761 (4)°	Prism, colourless
γ = 111.686 (4)°	0.30 × 0.10 × 0.10 mm
*V* = 618.0 (3) Å^3^	

### Data collection

**Table:**

Bruker APEXII CCD area-detector diffractometer	2292 independent reflections
Radiation source: fine-focus sealed tube	1572 reflections with *I* > 2σ(*I*)
graphite	*R*_int_ = 0.030
φ and ω scans	θ_max_ = 25.5°, θ_min_ = 2.6°
Absorption correction: multi-scan (*SADABS*; Bruker, 2001)	*h* = −9→9
*T*_min_ = 0.969, *T*_max_ = 0.989	*k* = −10→10
4891 measured reflections	*l* = −11→11

### Refinement

**Table:**

Refinement on *F*^2^	Primary atom site location: structure-invariant direct methods
Least-squares matrix: full	Secondary atom site location: difference Fourier map
*R*[*F*^2^ > 2σ(*F*^2^)] = 0.048	Hydrogen site location: inferred from neighbouring sites
*wR*(*F*^2^) = 0.150	H-atom parameters constrained
*S* = 1.07	*w* = 1/[σ^2^(*F*_o_^2^) + (0.0824*P*)^2^ + 0.0107*P*] where *P* = (*F*_o_^2^ + 2*F*_c_^2^)/3
2292 reflections	(Δ/σ)_max_ < 0.001
175 parameters	Δρ_max_ = 0.24 e Å^−^^3^
0 restraints	Δρ_min_ = −0.19 e Å^−^^3^

### Special details

**Table:**

Geometry. All esds (except the esd in the dihedral angle between two l.s. planes) are estimated using the full covariance matrix. The cell esds are taken into account individually in the estimation of esds in distances, angles and torsion angles; correlations between esds in cell parameters are only used when they are defined by crystal symmetry. An approximate (isotropic) treatment of cell esds is used for estimating esds involving l.s. planes.
Refinement. Refinement of F^2^ against ALL reflections. The weighted R-factor wR and goodness of fit S are based on F^2^, conventional R-factors R are based on F, with F set to zero for negative F^2^. The threshold expression of F^2^ > 2sigma(F^2^) is used only for calculating R-factors(gt) etc. and is not relevant to the choice of reflections for refinement. R-factors based on F^2^ are statistically about twice as large as those based on F, and R- factors based on ALL data will be even larger.

### Fractional atomic coordinates and isotropic or equivalent isotropic displacement parameters (Å^2^)

**Table:**

	*x*	*y*	*z*	*U*_iso_*/*U*_eq_	
C1	0.3325 (3)	1.0614 (2)	0.9868 (2)	0.0430 (5)	
C2	0.2915 (3)	1.0571 (3)	1.1282 (2)	0.0463 (5)	
H2	0.3657	1.1427	1.1938	0.056*	
C3	0.1515 (3)	0.9353 (2)	1.1690 (2)	0.0409 (5)	
C4	0.0343 (2)	0.8014 (2)	1.0672 (2)	0.0354 (5)	
C5	−0.1181 (3)	0.6703 (2)	1.0954 (2)	0.0408 (5)	
H5	−0.1486	0.6655	1.1852	0.049*	
C6	−0.2241 (3)	0.5482 (2)	0.9946 (2)	0.0419 (5)	
H6	−0.3251	0.4615	1.0156	0.050*	
C7	−0.1786 (2)	0.5559 (2)	0.8607 (2)	0.0368 (5)	
C8	−0.0288 (2)	0.6828 (2)	0.8251 (2)	0.0349 (5)	
C9	0.0747 (2)	0.8046 (2)	0.9306 (2)	0.0344 (5)	
C10	0.1127 (3)	0.9338 (3)	1.3169 (2)	0.0646 (7)	
H10A	0.2010	1.0299	1.3709	0.097*	
H10B	0.1145	0.8314	1.3574	0.097*	
H10C	−0.0023	0.9390	1.3168	0.097*	
C11	0.0155 (3)	0.6857 (2)	0.6786 (2)	0.0432 (5)	
C12	0.1973 (3)	0.6967 (3)	0.6594 (3)	0.0593 (7)	
H12A	0.1881	0.6088	0.5925	0.089*	
H12B	0.2594	0.6830	0.7475	0.089*	
H12C	0.2612	0.8052	0.6259	0.089*	
C13	−0.4432 (3)	0.4153 (3)	0.7065 (2)	0.0463 (5)	
C14	−0.5350 (3)	0.2647 (3)	0.6076 (3)	0.0634 (7)	
H14A	−0.6577	0.2494	0.5840	0.095*	
H14B	−0.5263	0.1661	0.6508	0.095*	
H14C	−0.4803	0.2813	0.5241	0.095*	
O1	0.21819 (16)	0.93313 (16)	0.89181 (14)	0.0416 (4)	
O2	0.4560 (2)	1.16541 (18)	0.94235 (18)	0.0601 (5)	
O3	−0.27782 (17)	0.42394 (16)	0.76069 (15)	0.0447 (4)	
O4	−0.5014 (2)	0.5178 (2)	0.73698 (19)	0.0684 (5)	
O5	−0.0952 (2)	0.6742 (2)	0.57939 (17)	0.0694 (5)	

### Atomic displacement parameters (Å^2^)

**Table:**

	*U*^11^	*U*^22^	*U*^33^	*U*^12^	*U*^13^	*U*^23^
C1	0.0312 (11)	0.0310 (11)	0.0618 (14)	0.0074 (9)	0.0020 (10)	0.0004 (10)
C2	0.0374 (12)	0.0396 (11)	0.0551 (14)	0.0110 (9)	−0.0060 (10)	−0.0082 (10)
C3	0.0402 (11)	0.0389 (11)	0.0420 (12)	0.0154 (9)	−0.0016 (9)	−0.0018 (9)
C4	0.0314 (10)	0.0343 (10)	0.0405 (11)	0.0132 (8)	0.0027 (8)	0.0019 (8)
C5	0.0393 (11)	0.0409 (11)	0.0417 (11)	0.0134 (9)	0.0093 (9)	0.0056 (9)
C6	0.0312 (10)	0.0360 (11)	0.0529 (13)	0.0047 (8)	0.0103 (9)	0.0043 (9)
C7	0.0292 (10)	0.0314 (10)	0.0463 (12)	0.0091 (8)	0.0000 (8)	−0.0022 (8)
C8	0.0296 (10)	0.0331 (10)	0.0421 (11)	0.0125 (8)	0.0034 (8)	0.0007 (8)
C9	0.0248 (9)	0.0308 (10)	0.0463 (12)	0.0090 (8)	0.0046 (8)	0.0050 (8)
C10	0.0727 (17)	0.0653 (16)	0.0455 (14)	0.0164 (13)	0.0016 (12)	−0.0083 (12)
C11	0.0415 (12)	0.0403 (11)	0.0456 (12)	0.0133 (9)	0.0050 (10)	−0.0001 (9)
C12	0.0539 (14)	0.0754 (16)	0.0571 (15)	0.0293 (12)	0.0221 (12)	0.0068 (12)
C13	0.0319 (11)	0.0500 (12)	0.0508 (13)	0.0092 (10)	0.0026 (9)	−0.0017 (10)
C14	0.0442 (14)	0.0689 (16)	0.0619 (16)	0.0076 (12)	−0.0036 (12)	−0.0196 (13)
O1	0.0315 (7)	0.0372 (8)	0.0489 (9)	0.0040 (6)	0.0070 (6)	0.0021 (6)
O2	0.0442 (9)	0.0414 (9)	0.0804 (12)	−0.0021 (7)	0.0141 (8)	0.0035 (8)
O3	0.0315 (8)	0.0380 (8)	0.0578 (9)	0.0075 (6)	0.0004 (6)	−0.0097 (7)
O4	0.0503 (10)	0.0742 (12)	0.0835 (13)	0.0333 (9)	−0.0093 (9)	−0.0180 (10)
O5	0.0583 (11)	0.1014 (14)	0.0466 (10)	0.0311 (10)	−0.0026 (8)	−0.0036 (9)

### Geometric parameters (Å, °)

**Table:**

C1—O2	1.204 (2)	C9—O1	1.374 (2)
C1—O1	1.383 (2)	C10—H10A	0.9600
C1—C2	1.445 (3)	C10—H10B	0.9600
C2—C3	1.338 (3)	C10—H10C	0.9600
C2—H2	0.9300	C11—O5	1.203 (2)
C3—C4	1.453 (3)	C11—C12	1.495 (3)
C3—C10	1.502 (3)	C12—H12A	0.9600
C4—C5	1.394 (3)	C12—H12B	0.9600
C4—C9	1.400 (3)	C12—H12C	0.9600
C5—C6	1.369 (3)	C13—O4	1.191 (2)
C5—H5	0.9300	C13—O3	1.361 (2)
C6—C7	1.388 (3)	C13—C14	1.483 (3)
C6—H6	0.9300	C14—H14A	0.9600
C7—C8	1.388 (3)	C14—H14B	0.9600
C7—O3	1.399 (2)	C14—H14C	0.9600
C8—C9	1.392 (3)	O2—C1^i^	3.058 (3)
C8—C11	1.504 (3)	O2—C13^ii^	3.101 (3)
			
O2—C1—O1	116.15 (19)	C3—C10—H10B	109.5
O2—C1—C2	126.87 (19)	H10A—C10—H10B	109.5
O1—C1—C2	116.97 (17)	C3—C10—H10C	109.5
C3—C2—C1	122.88 (19)	H10A—C10—H10C	109.5
C3—C2—H2	118.6	H10B—C10—H10C	109.5
C1—C2—H2	118.6	O5—C11—C12	121.1 (2)
C2—C3—C4	118.98 (19)	O5—C11—C8	120.33 (19)
C2—C3—C10	121.69 (19)	C12—C11—C8	118.50 (18)
C4—C3—C10	119.33 (19)	C11—C12—H12A	109.5
C5—C4—C9	117.28 (18)	C11—C12—H12B	109.5
C5—C4—C3	124.53 (18)	H12A—C12—H12B	109.5
C9—C4—C3	118.19 (18)	C11—C12—H12C	109.5
C6—C5—C4	121.83 (19)	H12A—C12—H12C	109.5
C6—C5—H5	119.1	H12B—C12—H12C	109.5
C4—C5—H5	119.1	O4—C13—O3	122.68 (19)
C5—C6—C7	118.96 (18)	O4—C13—C14	126.4 (2)
C5—C6—H6	120.5	O3—C13—C14	110.89 (18)
C7—C6—H6	120.5	C13—C14—H14A	109.5
C6—C7—C8	122.35 (18)	C13—C14—H14B	109.5
C6—C7—O3	119.13 (17)	H14A—C14—H14B	109.5
C8—C7—O3	118.30 (17)	C13—C14—H14C	109.5
C7—C8—C9	116.78 (18)	H14A—C14—H14C	109.5
C7—C8—C11	120.47 (17)	H14B—C14—H14C	109.5
C9—C8—C11	122.75 (17)	C9—O1—C1	121.70 (15)
O1—C9—C8	115.92 (17)	C1—O2—C1^i^	88.63 (13)
O1—C9—C4	121.25 (17)	C1—O2—C13^ii^	143.37 (15)
C8—C9—C4	122.81 (18)	C1^i^—O2—C13^ii^	118.73 (8)
C3—C10—H10A	109.5	C13—O3—C7	117.16 (14)
			
O2—C1—C2—C3	−179.5 (2)	C3—C4—C9—O1	−1.9 (3)
O1—C1—C2—C3	0.7 (3)	C5—C4—C9—C8	−0.8 (3)
C1—C2—C3—C4	−0.3 (3)	C3—C4—C9—C8	−179.87 (16)
C1—C2—C3—C10	179.75 (19)	C7—C8—C11—O5	50.4 (3)
C2—C3—C4—C5	−178.21 (18)	C9—C8—C11—O5	−129.3 (2)
C10—C3—C4—C5	1.7 (3)	C7—C8—C11—C12	−127.4 (2)
C2—C3—C4—C9	0.8 (3)	C9—C8—C11—C12	52.9 (3)
C10—C3—C4—C9	−179.22 (18)	C8—C9—O1—C1	−179.47 (15)
C9—C4—C5—C6	0.5 (3)	C4—C9—O1—C1	2.4 (3)
C3—C4—C5—C6	179.52 (17)	O2—C1—O1—C9	178.42 (16)
C4—C5—C6—C7	−0.2 (3)	C2—C1—O1—C9	−1.7 (3)
C5—C6—C7—C8	0.2 (3)	O1—C1—O2—C1^i^	−87.95 (16)
C5—C6—C7—O3	174.65 (16)	C2—C1—O2—C1^i^	92.2 (2)
C6—C7—C8—C9	−0.4 (3)	O1—C1—O2—C13^ii^	52.7 (3)
O3—C7—C8—C9	−174.95 (15)	C2—C1—O2—C13^ii^	−127.1 (2)
C6—C7—C8—C11	179.84 (18)	O4—C13—O3—C7	2.9 (3)
O3—C7—C8—C11	5.3 (3)	C14—C13—O3—C7	−177.22 (18)
C7—C8—C9—O1	−177.37 (14)	C6—C7—O3—C13	73.3 (2)
C11—C8—C9—O1	2.3 (3)	C8—C7—O3—C13	−112.06 (19)
C7—C8—C9—C4	0.7 (3)	C1—O2—C13^ii^—O4^ii^	69.0 (3)
C11—C8—C9—C4	−179.55 (17)	C1—O2—C1^i^—O2^i^	0.0
C5—C4—C9—O1	177.26 (15)		

Symmetry codes: (i) −*x*+1, −*y*+2, −*z*+2; (ii) *x*+1, *y*+1, *z*.

### Hydrogen-bond geometry (Å, °)

**Table:**

*D*—H···*A*	*D*—H	H···*A*	*D*···*A*	*D*—H···*A*
C6—H6···O2^iii^	0.93	2.52	3.324 (3)	145.

Symmetry codes: (iii) *x*−1, *y*−1, *z*.
